# Supplementary material for: Polyketide synthase gene clusters in the ericoid mycorrhizal fungus Oidiodendron maius: genome-wide prediction, expression profiling, and functional characterization of knockout mutants
Source: Front Plant Sci. 2026 Sep 16;17:1924430. doi: 10.3389/fpls.2026.1924430 (PMC13623903; doi:10.3389/fpls.2026.1924430)
Supplement: Supplementary file 1 [file SupplementaryFile1.zip › Supplementary Materials/Supplementary Folder 1.zip!/Oidma1_AssemblyScaffolds_Repeatmasked/knownclusterblast/cluster28/jgi.p_Oidma1_43670_mibig_hits.html]

| MIBiG Protein | Description | MIBiG Cluster | MiBiG Product | % ID | % Coverage | BLAST Score | E-value |
| --- | --- | --- | --- | --- | --- | --- | --- |
| CDM36724.1 | Cytochrome\_P450 | BGC0001360 | Mycophenolic\_acid\_biosynthetic\_gene\_cluster | 34 | 100.0 | 288 | 1.8e-77 |
| EED17999.1 | benzoate\_4-monooxygenase\_cytochrome\_P450,\_putative | BGC0000154 | Stipitatic\_acid\_biosynthetic\_gene\_cluster | 33 | 76.6859344894 | 209 | 1.1e-53 |
| EAL94097.1 | cytochrome\_P450\_monooxygenase,\_putative | BGC0000811 | Fumigaclavine\_C\_biosynthetic\_gene\_cluster | 40 | 32.9479768786 | 143 | 9.5e-34 |
| EAQ84780.1 | hypothetical\_protein | BGC0001219 | Shanorellin\_biosynthetic\_gene\_cluster | 33 | 41.0404624277 | 117 | 4.3e-26 |
| ADI24937.1 | VrtK | BGC0000168 | Viridicatumtoxin\_biosynthetic\_gene\_cluster | 24 | 97.6878612717 | 113 | 6.1e-25 |
| ctg1\_orf5 |  | BGC0000687 | Phyllocladan-16alpha-ol\_biosynthetic\_gene\_cluster | 33 | 28.1310211946 | 57 | 5.2e-08 |
| CAL58689.1 | cytochrome\_P450\_dependent\_monooxygenase | BGC0000149 | Spirangien\_biosynthetic\_gene\_cluster | 24 | 41.8111753372 | 55 | 2e-07 |
